# Hepatic artery pseudoaneurysm: three case reports and literature review

**DOI:** 10.3389/fmed.2024.1422895

**Published:** 2024-07-10

**Authors:** Yuan-Quan Zhao, Yong-Yu Yang, Si-Yang Yao, Xiao-Feng Dong

**Affiliations:** Department of Hepatobiliary, Pancreas and Spleen Surgery, The People’s Hospital of Guangxi Zhuang Autonomous Region, Nanning, China

**Keywords:** hepatic artery pseudoaneurysm, cholelithiasis, laparoscopic surgery, case report, transcatheter arterial embolization

## Abstract

Laparoscopic surgery is extensively applied in the treatment of hepatobiliary diseases. Hepatic artery pseudoaneurysm (HAP) is a rare complication following hepatic biliary surgery through laparoscopy. The clinical manifestations of HAP are diverse and can be fatal. Given its severity, rapid assessment and management are crucial to ensuring a good prognosis. Here, we report three cases of HAP; two underwent laparoscopic surgery due to cholelithiasis, and another caused by trauma. The first case exhibited a pseudoaneurysm involving the distal portion of the right hepatic artery main trunk. The second patient had a pseudoaneurysm at the bifurcation of the left and right hepatic arteries. The third case involved a patient with a pseudoaneurysm involving a branch of the right hepatic artery. The main clinical manifestations of all three cases were bleeding from the biliary tract (the first two cases showed postoperative bleeding in the T-tube, while the third case exhibited gastrointestinal bleeding). The final diagnosis was obtained through digital subtraction angiography. The three patients underwent successful transcatheter arterial embolization operation and a follow-up revealed they were disease-free and alive. This article aims to highlight a rare complication of laparoscopic hepatobiliary surgery and share our experience in early diagnosis and treatment of HAP.

## Introduction

Hepatic artery pseudoaneurysms (HAP) is considered a rare complication in patients undergoing surgical treatment for liver and gallbladder diseases through laparoscopic surgery (1, 2). Its actual incidence is difficult to estimate because many cases are asymptomatic or subclinical, or thrombus formation occurs spontaneously. The incidence of pseudoaneurysm after laparoscopic surgery is in the range of 0.06%–0.6%, mainly involving the hepatic artery and the residual end of the cystic artery (1, 3). Clinical reports indicate that HAP may occur in the initial days following laparoscopic surgery, or it may manifest weeks, months, or even years later (1, 4). Furthermore, HAP can also arise subsequent to trauma, inflammation, interventional procedures, and liver transplantation in patients (5, 6). Nowadays, HAP is increasingly being diagnosed in clinical practice, and this may be due to the increased use of laparoscopic biliary diagnostic and therapeutic procedures, as well as emergence of newer imaging techniques. However, there is no standard diagnostic procedure or treatment for HAP. Here, we report our experience with the diagnosis and treatment of three patients with HAP, hoping to provide assistance for the early identification, diagnosis, and treatment of HAP in the future.

## Case presentations

### Case 1

**FIGURE 1:**
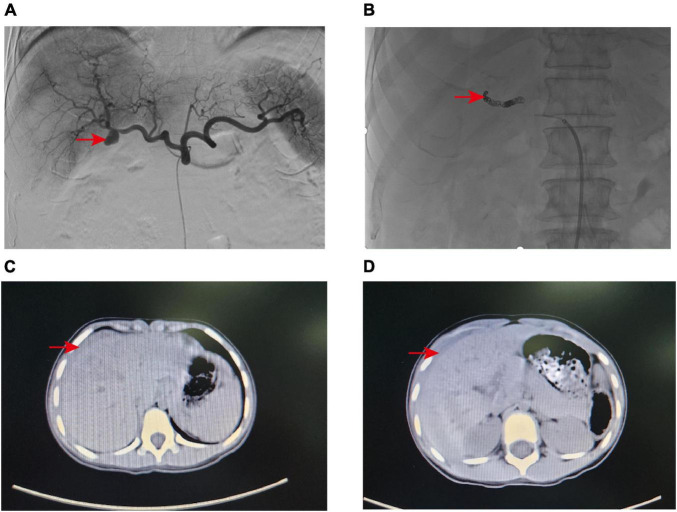
**(A)** Angiography revealed a pseudoaneurysm at the distal end of the right hepatic artery trunk. **(B)** The pseudoaneurysm was successfully embolized with Microcoils. **(C,D)** CT scan indicated that the pediatric patient had liver contusion with surrounding hematoma formation.

A 79-year-old male patient was admitted to the hospital as an emergency case due to bleeding from the T-tube with somnolence 2 weeks after undergoing laparoscopic surgery for cholelithiasis treatment. The patient has a history of type 2 diabetes and underwent lumbar surgery in 2018. A little over half a month ago, at our institution, the patient underwent laparoscopic common bile duct exploration with T-tube drainage and cholecystectomy. Routine blood tests showed Hb 62 g/L, and emergency computed tomography (CT) of the abdomen revealed an accumulation of blood around the T-tube, liver, and spleen, and accumulated fluid in the abdominal cavity. Subsequently, fasting, fluid replacement, blood transfusion, and hemostatic drugs (ethamsylate, tranexamic acid, and snake venom thrombin) were performed, however, the patient continued bleeding and underwent digital subtraction angiography (DSA), more precisely selective hepatic arteriography. This led to the identification of an isolated pseudoaneurysm, approximately 1.5 cm in diameter, originating distally from the main trunk of the right hepatic artery (approximately 1.0 cm from the bifurcation) (Figure 1A). In this case, to preserve the blood flow of the right hepatic artery and to avert the risk of fatal hepatic necrosis and hemorrhage consequent to the rupture of the pseudoaneurysm, the endovascular covered stent was prioritized for the treatment of HAP. Therefore, the endovascular covered stent placement was performed. A heparin-coated peritoneal stent (GORE, VBHR 050202W) was successfully placed distal to the right hepatic artery trunk and expanded using a 5 mm × 60 mm balloon (COOK, USA). Angiography reexamination demonstrated that contrast medium overflow could be seen at the distal end of right hepatic artery trunk, and arterial stent implantation failed. Subsequently, the patient underwent an emergent transcatheter arterial embolization (TAE) procedure. A microcatheter was advanced to the distal right hepatic artery, and Microcoils (COOK, USA) were deployed to embolize the pseudoaneurysm distally. Angiography confirmed successful embolization with no contrast flow into the pseudoaneurysm and normal right hepatic artery perfusion (Figure 1B). To prevent thrombosis after endovascular covered stent placement, the patient was administered anticoagulant therapy (specifically, Rivaroxaban 10 mg once daily) for a continuous period of 2 months, and antiplatelet therapy (specifically, Aspirin enteric-coated tablets 100 mg once daily) for a duration of 1 year.

Following TAE, in collaboration with the nursing team, we conducted a comprehensive postoperative care regimen for the patient, which included: (a) monitoring of vital signs such as body temperature, pulse, respiration, and blood pressure, with vigilant observation for symptoms such as fever, nausea, vomiting, and abdominal pain; (b) applying pressure dressings to the puncture site and immobilizing the limbs for 24 h, assisting the patient with turning and lateral positioning, and closely monitoring changes in the skin temperature, sensation, and color of the lower extremities; and (c) providing dietary guidance to the patient. The patient recovered rapidly within a few days and was discharged after biochemical parameters returned to normal. At the outpatient follow-up 1 month later, the patient was completely asymptomatic, with normal T-tube drainage of ink-green bile, no abdominal pain, melena, or jaundice, and normal liver function.

### Case 2

**FIGURE 2:**
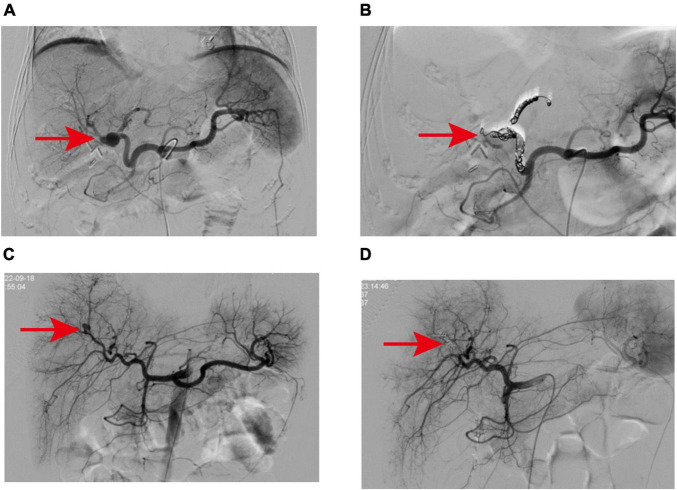
**(A)** The pseudoaneurysm was located at the bifurcation of the left and right hepatic arteries and showed multiple collateral circulation, which was related to the two branches of the left hepatic artery trunk and the right hepatic artery. **(B)** Microcoils were successfully used to embolize the vessels from the left hepatic artery to the neck of the aneurysm and from the branches of the right hepatic artery to the distal branches of the proper hepatic artery. **(C)** Angiography revealed a pseudoaneurysm in a branch of the right hepatic artery. **(D)** After embolization of pseudoaneurysms with Microcoils and embolic particles, the contrast extravasation disappeared.

A 57-year-old female patient was admitted to the hospital 4 weeks after undergoing laparoscopic surgery to treat cholelithiasis. The main complaint was T-tube bleeding 7 days after cholelithiasis surgery. The patient’s medical history indicates a prior lumbar surgery conducted in 2015, and 1 month prior, at our hospital, the patient underwent a laparoscopic common bile duct exploration with T-tube drainage and cholecystectomy. On admission, the patient had severe anemia (Hb 52 g/L), listlessness, and pale skin. Consequently, active blood transfusion, administration of hemostatic drugs (ethamsylate, tranexamic acid, and snake venom thrombin), and intravenous infusion were performed which slightly improve the patient’s symptoms. Initially, we suspected upper gastrointestinal bleeding and thus performed an upper gastrointestinal endoscopy which suggested chronic non-atrophic gastritis. After aggressive conservative treatment, the patient’s condition stabilized. On day 7 after admission, the patient experienced a sudden onset of hematemesis, darkred, with a volume of approximately 200 ml, accompanied by T-tube drainage of bloody fluid, abdominal pain, and fever (38.9°C). Emergency laboratory tests revealed a white blood cell count of 25.83 × 10^9^/L, Hb 64 g/L, and the patient received blood transfusions, gastrointestinal decompression, anti-infection, intravenous infusion, among other interventions. A blood routine examination the next day showed a cell count of 9.35 × 10^9^/L and Hb 68 g/L, however, the patient showed bright red bloody fluid outflow from the T-tube. Based on these findings, we suspected that the patient still had active hemorrhage. Subsequently, we performed an emergency elective hepatic arteriography on the patient. A 2.0 cm solitary pseudoaneurysm with aneurysmal dilation was identified at the confluence of the left and right hepatic arteries. Extensive collateral circulation surrounded the left hepatic artery trunk and the two branches of the right hepatic artery (Figure 2A). Further analysis showed that the endovascular covered stent placement was not suitable for this patient because the HAP was present at the intersection of the left and right hepatic arteries, there were multiple tortuous collateral vessels, and its size was not appropriate for the currently marketed covered stent. Therefore, we performed TAE on patients. Multiple tortuous collateral vessels were successfully embolized using Microcoils (COOK, USA) (Figure 2B). Following TAE, with the support of our nursing team, we provided the patient with comprehensive postoperative care akin to that administered in case 1. Ultimately, all biochemical indicators of the patient returned to normal, and the patient was discharged after the T-tube was successfully removed. During the outpatient follow-up visit 1 month postoperatively, the patient was disease-free and in good condition.

### Case 3

A 6-year-old girl was admitted to the emergency department due to abdominal pain after trauma. The patient’s medical history is unremarkable. The patient had no bloody stool or hematuria at admission, and a CT examination performed immediately revealed a possible formation of liver contusion and perihepatic hematoma (Figures 1C, D). After symptomatic support treatment, the child’s condition gradually improved. After 6 days of admission, she developed suddenly hematemesis, melena, and hemorrhagic shock. Thus, she was transferred to the pediatric intensive care unit for further treatment. Blood transfusion was conducted and hemostatic drugs were prescribed. The gastrointestinal endoscopy was performed immediately and suggested the possibility of blood pooling in the intestinal lumen of the small intestine. To determine the cause of bleeding, we subsequently performed selective hepatic arteriography. The right hepatic artery branch was found to be damaged and a significant pseudoaneurysm was formed (Figure 2C), measuring about 1.2 cm in diameter, and contrast medium overflow was detected. Subsequently, we performed emergency TAE, successfully embolizing the feeding branch of the HAP with Microcoils (COOK, USA) and embosphere microspheres (Figure 2D). Following TAE, we provided the child with comprehensive postoperative care tailored to her condition, akin to the care administered in case 1. One week post-procedure, the patient’s blood routine and liver function indices returned to normal, facilitating a smooth discharge. Upon outpatient follow-up 1 month later, the child exhibited no adverse symptoms and maintained normal blood routine and liver function.

## Discussion

Hepatic artery pseudoaneurysm is a rare complication associated with laparoscopic surgery which can lead to life-threatening bleeding and infection (1, 2). The complication may also occur following trauma, inflammation, percutaneous interventions, and liver transplantation patients (5, 6). HAP is usually asymptomatic if there is no rupture hemorrhage or infection. Hemobilia is the most common symptom of HAP, and the classic Quincke triad includes upper abdominal pain, jaundice, and hemobilia (often manifesting as gastrointestinal bleeding), occurring in up to one-third of patients (7). In this report, two patients (case 1 and case 2) developed HAP after laparoscopic cholecystectomy and choledocholithotomy, presenting primarily with hemobilia (T-tube hemorrhage). In another patient (case 3), experienced HAP shortly after an abdominal trauma and presented with gastrointestinal bleeding (hematemesis and melena). The portal triad, which includes the portal vein, hepatic artery, and bile duct, converges at the hepatic hilum to enter the liver. However, its development is not simultaneous during embryonic stages. At 8 weeks gestation, a bile duct plate forms at the liver hilum, extending along the portal vein. Between 12 weeks and birth, this plate is reshaped, with the hepatic artery accompanying its growth. Blood vessels from the hepatic artery eventually supply the mature biliary system (8, 9). This may form the anatomical basis for HAP to easily rupture into bile ducts and cause hemobilia and gastrointestinal hemorrhage.

The occurrence of HAP is driven by several injury mechanisms, including tearing, transection, or occlusion of vessels following mechanical or thermal injury to arteries. It may also be triggered by inappropriate energy (thermal/mechanical) application during or after dissection, such as calot triangle dissection, choledochoscopy on the common bile duct, T-tube suturing, and fixation. Thermal damage can occur through heat transfer directly to the blood vessels by cauterization or indirectly through metal clips in contact with the arteries (10). Clamp intrusion, bile leakage, or infection may also lead to arterial damage, leading to HAP (4, 11). Infectious bile from bile leakage is thought to cause direct weakening and erosion of the blood vessel wall, thus increasing the risk of HAP (12, 13). Infection may further promote the thinning of the bile duct wall and rupture of HAP into the biliary tract system. Because secondary infection may accelerate the development of HAP in patients with biloma (14). CO_2_ pneumoperitoneum, which increases intra-abdominal pressure, has been shown to significantly reduce portal venous blood flow compared to hepatic arterial blood flow (15). This hemodynamic change causes fluctuating blood flow at the bifurcation and instability in hepatic artery pressure, leading to vortex generation. Hemodynamic abnormalities are major contributors to vascular disease and may also lead to HAP (16, 17). In general, the pathogenesis of HAP is not well understood and needs to be further explored. Therefore, timely and accurate diagnosis as well as treatment of HAP are particularly critical.

The diagnosis of HAP can be confirmed through a combination of medical history (surgical history, trauma history, and inflammatory), upper gastrointestinal endoscopy, ultrasound, contrast CT scans, and/or DSA (1, 6). Among these approaches, selective hepatic arteriography is the most accurate diagnostic method for HAP (18) and is employed in treating HAP. It can visualize the injured arterial wall, and if the arterial wall is broken or ruptured, the contrast medium will overflow. Hence, it can be used to assess the rupture and feeding artery of HAP, and appropriate embolization materials such as occlusive balloon, gelatin sponge, and Microcoil can be utilized for minimally invasive interventional treatment. More importantly, selective hepatic arteriography can rule out conditions unsuitable for interventional treatment, including HAP with numerous tortuous collateral vessels and HAP with a wide neck and extensive collateral circulation. In all three of our patients, selective hepatic arteriography provided the definitive diagnosis and enabled treatment.

Hepatic artery pseudoaneurysm is considered an acute emergency that require immediate and aggressive intervention because rupture can lead to life-threatening hemorrhagic shock. The risk of rupture in HAP patients is 21%–80%, and the incidence of death due to rupture bleeding is approximately 43% (1). In recent years, with the development of minimally invasive techniques, HAP is primarily treated by interventional methods, including TAE and endovascular covered stent placement, while surgery is only used when it is the only option. Among these measures, TAE is the best way to manage HAP. Compared with surgery, interventional therapy is less invasive, safer, and has a higher success rate. However, arteriographic embolization may be associated with significant risks, including the rupture of the HAP during coil embolization, misplacement of embolic agents leading to non-target vessel occlusion, hepatic ischemic necrosis, hemorrhage, abscess formation, and biliary stricture due to ischemia (1, 15, 19). Consequently, it is imperative to assess adequate arterial and collateral blood flow prior to hepatic artery embolization to mitigate the risk of localized ischemia and prevent complications associated with reduced blood flow. Of note, interventional therapy may not be appropriate for some patients, such as those who cannot undergo TAE due to the wide neck and multiple collateral feeding of the HAP or those who do not have suitable covered stents for endovascular covered stent placement as a result of multiple tortuous collateral vessels (20, 21). In some cases, TAE embolization failures may also make access to HAP difficult due to the formation of thrombus in proximal vessels (11). Even after successful hepatic artery catheterization, TAE embolization can fail due to several factors. These include failing to isolate the bleeding vessels, misidentifying the source of bleeding, or incomplete occlusion despite the absence of visible sacs or feeding vessels on post-embolization angiography (20, 22). For HAP patients who fail embolization, non-selective embolization or emergency surgery may be performed, but large-area non-selective embolization or surgical removal of excess liver tissue may lead to liver failure risk (1). In one of our patients (case 2), HAP was located at the intersection of the left and right hepatic arteries and had multiple collateral circulation, involving the main left hepatic artery and two branches communicating with the right hepatic artery. Endovascular covered stent placement was not suitable for this patient, thus, TAE was selected and embolization was successful. Selective hepatic arteriography and successful embolization were achieved in the remaining two patients with HAP who lacked significant collateral supply, vessel flexion, or proximal vessel thrombosis.

## Conclusion

Hepatic artery pseudoaneurysm is an emergency and life-threatening condition, and timely diagnosis and treatment are crucial. The diagnosis of HAP requires comprehensive examination of the medical history, clinical manifestations, and auxiliary examinations. Doppler ultrasound, CT scan, and endoscopic examinations of the gastrointestinal tract can be performed as auxiliary diagnostic methods for HAP as they provide meaningful clues and help differential diagnosis. Selective hepatic arteriography is the gold standard for imaging diagnosis of HAP, because it can accurately display the shape, location and collateral feeding arteries of the tumor, and provide minimally invasive interventional therapy. TAE is the preferred treatment for HAP, and endovascular covered stent placement is also commonly used in clinical practice. Surgery should be conducted as a second-line treatment for HAP in patients with failed TAE and endovascular covered stent placement to save lives.

## Data availability statement

The original contributions presented in this study are included in this article/supplementary material, further inquiries can be directed to the corresponding author.

## Ethics statement

The studies involving humans were approved by the Ethics Committee of the People’s Hospital of Guangxi Zhuang Autonomous Region. The studies were conducted in accordance with the local legislation and institutional requirements. Written informed consent for participation was not required from the participants or the participants’ legal guardians/next of kin in accordance with the national legislation and institutional requirements. Written informed consent was obtained from the individual(s) for the publication of any potentially identifiable images or data included in this article.

## Author contributions

Y-QZ: Data curation, Formal analysis, Investigation, Resources, Writing – original draft. Y-YY: Data curation, Formal analysis, Resources, Writing – original draft. S-YY: Data curation, Formal analysis, Investigation, Writing – original draft. X-FD: Funding acquisition, Supervision, Validation, Writing – review & editing.
